# Evaluation of the Control Options of Bovine Tuberculosis in Ethiopia Using a Multi-Criteria Decision Analysis

**DOI:** 10.3389/fvets.2020.586056

**Published:** 2020-12-16

**Authors:** Fanta D. Gutema, Getahun E. Agga, Kohei Makita, Rebecca L. Smith, Monique Mourits, Takele B. Tufa, Samson Leta, Tariku J. Beyene, Zerihun Asefa, Beksissa Urge, Gobena Ameni

**Affiliations:** ^1^College of Veterinary Medicine and Agriculture, Addis Ababa University, Bishoftu, Ethiopia; ^2^U. S. Department of Agriculture, Agricultural Research Service, Food Animal Environmental Systems Research Unit, Bowling Green, KY, United States; ^3^Department of Veterinary Medicine, School of Veterinary Medicine, Rakukno Gakuen University, Ebetsu, Japan; ^4^Department of Pathobiology, College of Veterinary Medicine, University of Illinois Urbana-Champaign, Urbana, IL, United States; ^5^Business Economics Group, Wageningen University, Wageningen, Netherlands; ^6^Department of Preventive Veterinary Medicine, Ohio State University, Columbus, OH, United States; ^7^Ethiopian Institute of Agricultural Research, Addis Ababa, Ethiopia; ^8^Aklilu Lemma Institute of Pathobiology, Addis Ababa University, Addis Ababa, Ethiopia; ^9^Department of Veterinary Medicine, College of Agriculture, United Arab Emirates University, Al Ain, United Arab Emirates

**Keywords:** bovine tuberculosis, multi-criteria decision analysis, stakeholders, control, Ethiopia

## Abstract

Bovine tuberculosis (BTB) is a zoonotic bacterial infection caused by *Mycobacterium bovis* and is characterized by the development of granulomatous lesions in the lymph nodes, lungs and other tissues. It poses serious public health impacts and food security challenges to the agricultural sector in terms of dairy and meat productions. In Ethiopia, BTB has been considered as a priority disease because of its high prevalence in urban and peri-urban dairy farms. However, there has not been any national control program in the country. Thus, in order to initiate BTB control program in the country, information on control options is needed to tailor the best option for the Ethiopian situation. The objective of this study was to identify, evaluate and rank various BTB control options in Ethiopia using a multi-criteria decision analysis based on preference ranking organization method for enrichment evaluations (PROMETHEE) approach while accounting for the stakeholders' preferences. Control options were evaluated under two scenarios: with (scenario 1) and without (scenario 2) bacillus Calmette–Guérin (BCG) vaccination. Nine potential control options were identified that include combinations of three control options (1) test and slaughter with or without government support, (2) test and segregation, and (3) BCG vaccination. Under scenario 1, BCG vaccination, BCG vaccination and test and slaughter with partial compensation by government, and BCG vaccination and test and slaughter with full compensation by government were the top three ranked control options. Under scenario 2, test and slaughter with full compensation by government was the preferred control option, followed by test and segregation supported by test and slaughter with full government compensation, and test and slaughter with half compensation by government. Irrespective of the variability in the weighting by the stakeholders, the sensitivity analysis showed the robustness of the ranking method. In conclusion, the study demonstrated that BCG vaccination, and test and slaughter with full compensation by government were the two most preferred control options under scenarios 1 and 2, respectively. National level discussions were strongly recommended for further concretization and implementation of these control measures.

## Introduction

BTB is a zoonotic bacterial infection caused by *M. bovis*, a member of the *Mycobacterium tuberculosis* complex (1). It causes a serious public health impact and food security and safety challenges (2). Contaminated dairy products are the main sources of BTB infections in humans, mainly resulting in extra-pulmonary infections such as lymphadenitis (3). According to the World Health Organization, there were 147,000 new cases of zoonotic TB and 12,500 human TB related deaths in 2016 with higher incidence and death rates in Africa than other parts of the world (4). Even though there are no comprehensive studies to estimate the global socio-economic costs of BTB, it causes significant economic losses due to production losses such as reduced milk yield, cost of surveillance and control programs and trade barriers with a major impact on the livelihoods of poor and marginalized communities (5).

In high income countries, public health risk and economic loss associated with *M*. *bovis* were considerably reduced or eliminated through the implementation of strict test-and-slaughter and meat inspection protocols for cattle, milk pasteurization, financial compensation to farmers and public education (6, 7). However, in most low and middle income countries where BTB is endemic, like in Ethiopia, such measures are hampered by financial constraints particularly for farmer compensation, and by inadequate veterinary services (8). Currently, there are several ongoing efforts to address zoonotic BTB to end the global TB epidemic by 2030 globally (4). However, there are no policies and implementation activities aligning to this global endeavor in the control of BTB in Ethiopia.

The conventional disease prevention and control interventions can have important environmental, social and economic impacts (9). For instance, test and slaughter policy is effective for control of BTB (10). However, it has several impacts such as killing large numbers of test positive animals, raising welfare concerns, and incurring costs for testing and compensation to cattle owners, making it economically difficult to apply particularly in resource limited countries. As a result, decision-making requires systems approach to integrate these multiple aspects of interventions. Multi-criteria decision analysis (MCDA) is an important and effective emerging system approach that can increase the understanding, acceptability and robustness of a decision problem of controlling zoonotic TB considering an integration of epidemiologic, economic and social-ethics value judgments (9, 11).

BTB was reported from 55% of herds and 32.3% of cattle in urban and peri-urban dairy farms in central Ethiopia (12). The national BTB prevalence estimate was 5.8% in individual cattle, with higher prevalence of 21.6% in exotic breeds and their crosses and 16.6% in herds kept under intensive and semi-intensive production systems in urban and peri-urban areas (13). Thus, it is particularly a problem for intensive dairy systems that raise dairy cattle with improved breed. For example, in the years 2005–2011, the maximum production loss due to BTB was estimated at $4.9 million in the urban livestock production systems in Ethiopia (14). Currently, there are no national policies and strategies for the control of BTB although the disease is considered among the top three diseases in dairy producing urban and peri-urban areas of the country in terms of prevalence and household impact (15). Researchers have recommended implementation of control in intensive and semi-intensive dairy farms due to the public health importance of BTB and concerns about spreading the disease through dairy cattle trade from the high prevalence urban system to low prevalence sedentary rural production systems (13, 15). To that end, information for decision making is needed to select and implement control options that are optimally tailored to the country's situations by considering the interests of the dairy farmers and the government. The objective of this study was to identify, evaluate and rank various BTB control options using a multi-criteria decision analysis tool. The outcome of the study would ultimately inform decision makers toward policy formulation and national level discussion to implement BTB control under semi-intensive and intensive dairy farming systems in Ethiopia.

## Materials and Methods

### Assembling Team

The study was conducted between July 2018 and June 2019. A multidisciplinary research team composed of researchers in the fields of veterinary public health, public health, veterinary epidemiology, infectious disease modeling, veterinary animal health economics, biostatistics and multi criteria decision analysis was assembled and involved in the study.

### Multi-Criteria Decision Analysis (MCDA)

The comprehensive and stepwise consecutive approaches of MCDA tool for managing zoonotic diseases as developed by Aenishaenslin et al. (9) was used to identify, evaluate and rank various BTB prevention and control options according to stakeholders' preferences to indicate the potential BTB control option under Ethiopian conditions. The approach consists of ten steps that were categorized into seven problem structuring and three decision analysis steps. In the context of the present study, stakeholders refer to key players in the control of BTB include representatives of governmental organizations, animal health professionals, public health professionals and experts (11).

### Problem Structuring

The problem structuring step consisted of the following steps: define the problem, identify the stakeholders, identify key decision issues, define criteria and indicators, identify intervention options, evaluate performance of each intervention option and weight criteria. Before conducting the MCDA, literature review was conducted on the available success stories on BTB controls in other countries to identify different control options. A non-systematic literature review approach was followed to search for focused available information on BTB control options using search engines such as Google scholars and PubMed with key phrases like “control of BTB,” “control of TB in cattle” and “control of zoonotic TB.” The generated articles were read in-depth for the targeted information and the citations in the articles were further referred when deemed necessary. Moreover, experts working on BTB in the academia and veterinary and medical government offices were consulted through face to face discussions and Skype meeting using check list of the important elements of the problem structuring steps of MCDA. Accordingly, they were consulted to contextualize the key decision issues related to BTB control in Ethiopia in terms of the prevalence of BTB in intensive and semi-intensive dairy farms, the need for BTB control, potential control options and measurements for the evaluation of the control options.

The actual MCDA analysis was performed through an interactive group discussion for which key stakeholders (*n* = 15) from various pertinent organizations in Ethiopia were invited. Out of the 15 stakeholders invited, 10 of them agreed to participate in the MCDA process (Table 1). The participating stakeholders conducted thorough interactive discussions to lay out problem structuring phase of the analysis, such as defining the problem and identifying key decision issues, defining the measurement scale on criteria, listing potential BTB control options, and evaluating the control options. The stakeholders identified 10 specific criteria (C1–C10) categorized into six clusters, namely epidemiology (1 criterion), practical applicability (1), economics (3), social ethics (3), public health (1) and animal welfare (1). All criteria were categorical ordinal variables (Table 2). Each stakeholder independently weighted each of the identified clusters and criteria and evaluated the performance of each of the control options based on three-point qualitative scale as low, medium or high (9).

**Table 1:** Composition of the stakeholders participated in the multi-criteria decision analysis for the evaluation of bovine tuberculosis control options in Ethiopia.

**Organizations**	**Number of participants**
Ministry of Agriculture	1
Ethiopian Commercial Dairy Producers Association	1
Ethiopian Public Health Institute	1
Adama General Hospital and Medical College	1
Ethiopian Meat and Dairy Industry Development Institute	1
Addis Ababa University, Pathobiology Institute	1
Addis Ababa University, College of Veterinary Medicine and Agriculture	2
Debre Berhan University	1
Ethiopian Institute of Agricultural Research Center	1

**Table 2:** Criteria used in the evaluation of bovine tuberculosis control options in Ethiopia.

**Criteria cluster**	**Brief description of criterion**
Epidemiology (EPI)	Reduction in BTB incidence or prevalence (C1)
Practical applicability (PA)	Level of difficulty in implementing the control option under Ethiopian condition (C2)
Economics (ECO)	Cost to the farmers -cost of test, loss of milk, replacement cost and other related costs (C3)
	Cost to the government- compensation of slaughtered animals, cost of laboratory test, veterinary costs (cost of farm visit, administrative cost to implement the control option (C4)
	Cost to the industry- inadequate milk supply to processing industries (C5)
Social ethics (SOE)	Acceptability by the Government (C6)
	Acceptability by dairy farmers (C7)
	Social impact - social crisis as a result of the intervention in terms of loss of high performing cow or loss of employment for labor workers (C8)
Public health (PH)	Public health impact - exposure to bovine tuberculosis during implementation of the intervention (C9)
Animal welfare (AW)	Impact on animal - welfare problems as a result of the intervention like slaughtering positive cattle, stress during vaccination and segregation (C10)

### Decision Analysis

The decision analysis step included: constructing a matrix based on multi-criteria analysis, sensitivity analysis and interpretation of the results. Since two differing opinions were made during the discussion by the stakeholders regarding inclusion and exclusion of BCG vaccination of calves as a control option under Ethiopian conditions, the comparison and option ranking were performed under two scenarios. Scenario 1 was modeled with the inclusion of BCG vaccination while scenario 2 was modeled by excluding it. The academic version of preference ranking organization method for enrichment evaluations (PROMETHEE) software 1.4 was used to perform pair-wise comparisons of the performance of control options using the preferences of the stakeholders to compute the overall outranking scores (16).

Based on the scores, the identified control options were listed from the most to the least preferred option. For ranking of the control options and visual display of the analysis results, the Geometrical analysis for interactive aid (GAIA) with two dimension (U-V) views and PROMETHEE table were used. The action profiles of the control options were performed for the top ranked control options to evaluate their relative performance on each criterion. The GAIA and sensitivity analysis were performed for scenario 1. GAIA walking weights were run to conduct sensitivity analysis to see the effect of weighing the evaluation criteria by stakeholders on the group ranking when the weights of the criterion were changed and to assess the robustness of the results.

## Results

From the 10 stakeholders participate in the MCDA process (S1–S10), nine were from the government organization and one stakeholder represented an association of privately owned dairy farmers (Table 1). The stakeholders agreed that BTB is a major problem and that the prevalence is particularly high in dairy herds with exotic cattle breeds and their crosses kept under semi intensive or intensive production system in urban and peri-urban dairy farming. They also emphasized the need for pooling collective efforts toward the control of BTB in the country targeting intensive and semi-intensive dairy farms in urban and peri-urban areas, noting the lack of national BTB control or eradication program in Ethiopia. The research team and stakeholders indicated the occurrence of high prevalence of BTB in semi-intensive and intensive dairy farms and the need for designing and implementing potential control option. The stakeholders identified nine possible control options including combinations of three specific options: test and slaughter with or without financial compensation, test and segregation, and BCG vaccination, with the assumption that each option can be implemented independently (Table 3). For effective implementation of BTB control option in the country, the stakeholders emphasized also the need for stringent prerequisites such as legal framework for implementation, preliminary BTB status testing of each animal and herd, animal identification and animal movement control, biosecurity measures at dairy farms, public education and BTB herd certification as supplementary/complementary measures to the implementation of potential intervention option(s).

**Table 3:** Descriptions of single and combined control options identified by stakeholders for control of bovine tuberculosis (BTB) in Ethiopia.

**BTB control option**	**Description**
BCG vaccination (OP1)	Calf vaccination with BCG vaccine at 6 weeks of age.
Test and segregation (OP2)	Testing and segregating infected animals at early stage of the disease and calf at birth, and switching to test-and-slaughter method in the final stage.
Test and slaughter with cost sharing (OP3)	Testing and slaughtering positive animals with the government and the owner equally sharing the cost of compensation for the slaughtered animals.
Test and slaughter with government support (OP4)	Testing and slaughtering positive animals with the government compensating full cost of the slaughtered animals to the owner.
BCG vaccination and test and segregation (OP5)	Calf vaccination to reduce the prevalence of the disease in the herd, and switch to test and segregation.
BCG vaccination and test and slaughter with cost sharing (OP6)	Calf vaccination and switching to testing and slaughtering of positive animals with the government and the owner equally sharing the cost of compensation for the slaughtered cattle.
BCG vaccination and test and slaughter with government support (OP7)	Calf vaccination, and switch to testing and slaughtering of positive animal with the government compensating full cost of the slaughtered animals to the owner.
Test and segregation and test and slaughter with cost sharing (OP8)	Testing and segregating infected animals, and switch to testing and slaughtering positive animals with the government and the owner equally sharing the cost of compensation for the slaughtered animals.
Test and segregation and test and slaughter with government support (OP9)	Testing and segregating infected animals, and switch to testing and slaughtering positive animals with the government compensating full cost of the slaughtered animals to the owner.

* *BTB test refers to application of tuberculin skin test that consists of injecting bovine tuberculin, a purified protein extract derived from M. bovis, intradermally and measuring the skin thickness at the site of injection after 72 h to detect any subsequent swelling at the injection site-sign of delayed hypersensitivity reaction associated with infection*.

**Figure 1:**
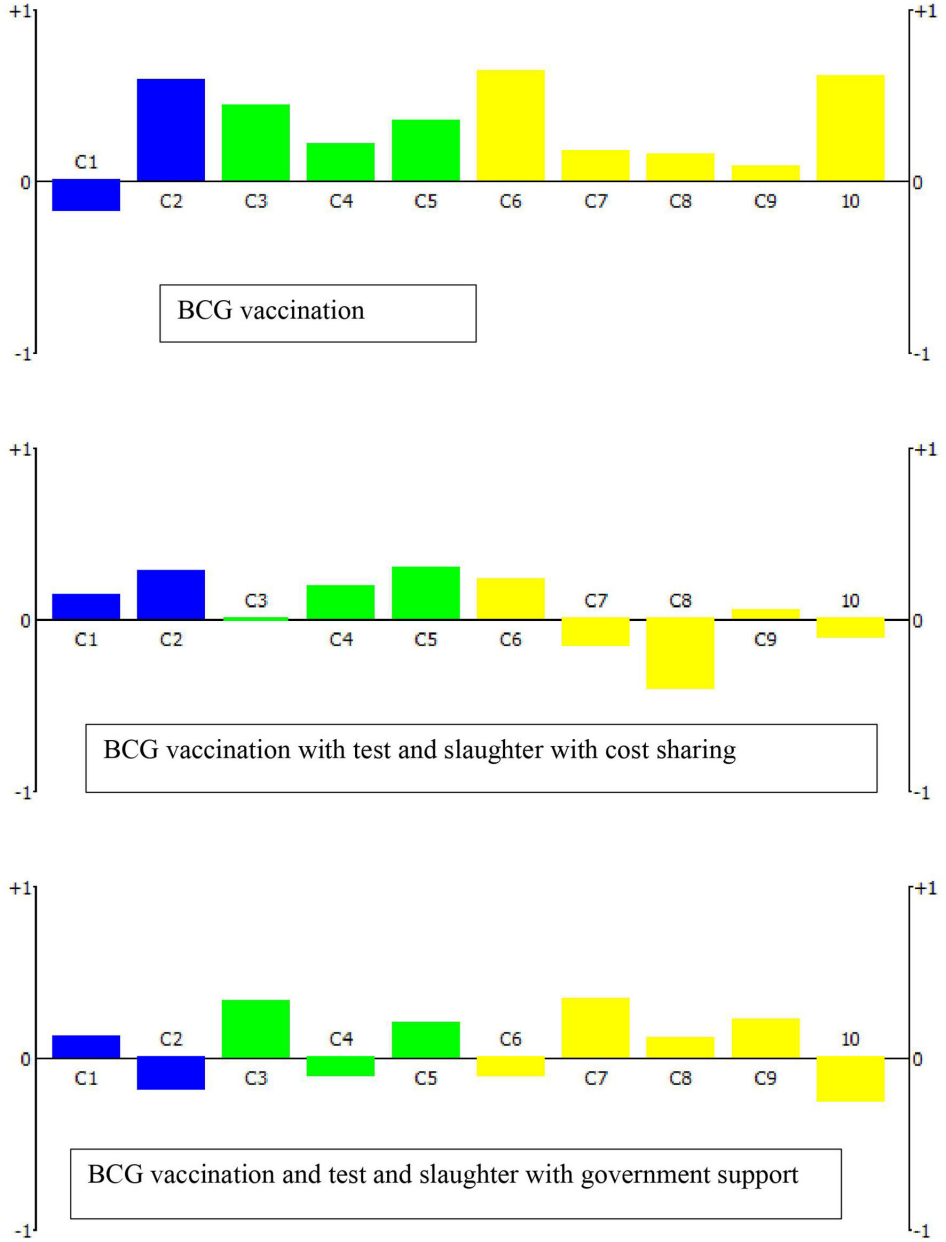
The action profiles of the top three control options under scenario 1 (use of BCG vaccination) indicating the net score per decision criterion (C1–C10; see Table 2). A score of −1 means that the evaluated control option has the worst performance for this indicator among all alternatives, while a score of +1 indicates the best performance.

All stakeholders generated a specific weighting scheme based on their perceived relative importance of each criterion as defined for the intended decision-making process. For this, each stakeholder was provided 100 points, and was asked to distribute the points to all specified criteria. The weighting of the stakeholders varied among the clusters and within the cluster criteria. Three stakeholders (S2, S3, and S10) gave the highest weight for the economic criteria, while the other seven stakeholders gave the highest weight to the epidemiologic cluster: reduction of BTB prevalence. The social ethics cluster and criteria generally received the least weight by all stakeholders (Table 4). Under scenario 1, the ranking of the control options showed that BCG vaccination (OP1), BCG vaccination combined with test and slaughter with cost sharing (OP6), and BCG vaccination combined with test and slaughter with full compensation of the cost by the government (OP7) as the top three potential control options. Figure 1 shows the relative performance of the top three control options on each criterion. The first ranked option, BCG vaccination, performs well on C2, C6, and C10 while poorly performing on C1 i.e., reduction in the prevalence/incidence of BTB. Conversely, the second and the third control options relatively perform well on C1 while poorly performed significantly on C8 and C10, respectively. Under scenario 2, test and slaughter with full government compensation (OP4) was the preferred control option followed by test and segregation combined with test and slaughter with full government compensation (OP9), and test and slaughter with half compensation by government (OP3) (Table 5).

**Table 4:** The relative weight given by stakeholders based on their preference for each cluster and specific criteria for the control of bovine tuberculosis (BTB) in Ethiopia.

**Cluster**	**Criteria**	**Weights**									
		**S1**	**S2**	**S3**	**S4**	**S5**	**S6**	**S7**	**S8**	**S9**	**S10**
Epidemiology	Reduction in BTB incidence (C1)	**25**	**20**	**20**	**30**	**25**	**25**	**25**	**25**	**30**	**20**
Practical applicability	Practical applicability (C2)	**15**	**10**	**10**	**15**	**15**	**15**	**15**	**15**	**20**	**10**
Economics	Cost to the farm owner (C3)	10	15	20	10	15	15	15	15	10	20
	Cost to the government (C4)	10	10	15	9	10	10	10	10	10	20
	Cost to the industry (C5)	10	15	15	6	10	10	10	10	10	10
	**Sub total**	**30**	**40**	**50**	**25**	**35**	**35**	**35**	**35**	**30**	**50**
Social ethics	Acceptance by government (C6)	6	10	5	5	5	5	5	5	5	5
	Acceptance by owner (C7)	4	5	5	5	5	5	5	5	5	5
	Social impact (C8)	4	5	5	4	5	5	5	5	5	2
	**Sub total**	**14**	**20**	**25**	**14**	**15**	**15**	**5**	**15**	**15**	**12**
Public health	Public health impact (C9)	**10**	**6**	**5**	**10**	**5**	**5**	**7**	**5**	**3**	**3**
Animal welfare	Impact on animal welfare (C10)	**6**	**4**	**5**	**6**	**5**	**5**	**3**	**5**	**2**	**5**
	**Total**	**100**	**100**	**100**	**100**	**100**	**100**	**100**	**100**	**100**	**100**

*The bold numbers indicate the weight given for each cluster out of 100 points by each stakeholder*.

**Table 5:** Group ranking for bovine tuberculosis control options in Ethiopia.

**Alternative control option**	**Scenario 1**		**Scenario 2**	
	**Score**	**Rank**	**Score**	**Rank**
BCG vaccination (OP1)	0.250	1	NA	NA
BCG vaccination, and test and slaughter with government support (OP7)	0.111	2	NA	NA
BCG vaccination, and test and slaughter with cost sharing (OP6)	0.071	3	NA	NA
Test and slaughter with government support (OP4)	0.006	4	0.100	1
BCG vaccination, and test and segregation (OP5)	−0.015	5	NA	NA
Test and segregation, and test and slaughter with government support (OP9)	−0.068	6	0.019	2
Test and slaughter with cost sharing (OP3)	−0.074	7	0.018	3
Test and segregation (OP2)	−0.141	8	−0.070	5
Test and segregation, and test and slaughter with cost sharing (OP8)	−0.140	9	−0.067	4

*NA, not applicable*.

**Figure 2:**
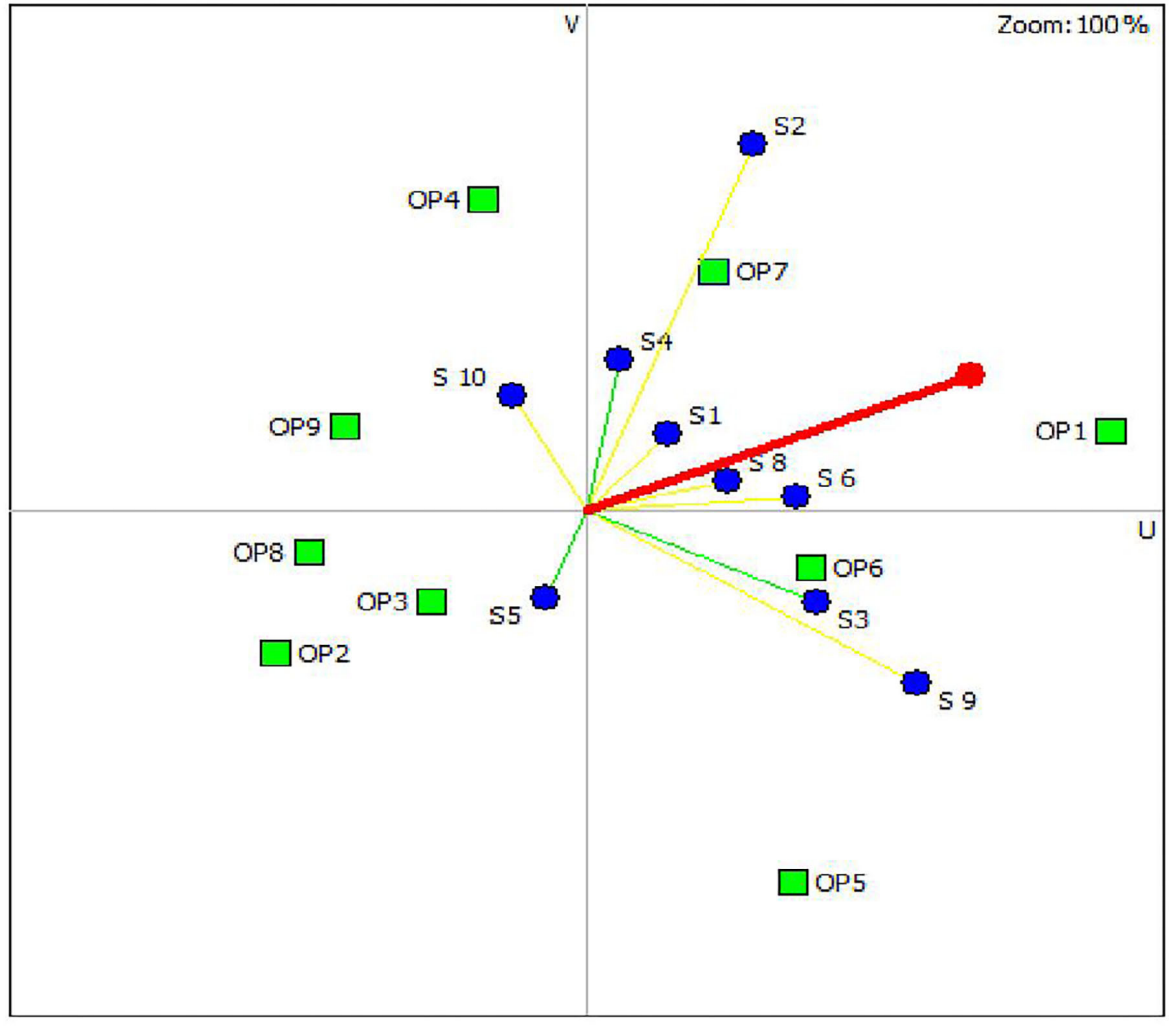
Geometrical analysis for interactive aid decision map for the intervention options under scenario 1 involving BCG vaccination (Delta = 71%, meaning that 71% of the information is conserved in the two-dimensional plane).

Figure 2 is the GAIA-scenarios plane visually displaying the positions of the control options and the stakeholders' preferences for scenario 1. As indicated in the GAIA plane, the options OP1, OP7, and OP6 are located on the right positions close to the decision axis representing the preferred control options while OP2 and OP8 are positioned in the left away from the decision axis representing the less preferred options that agreed with the results generated by the PROMETHEE table (Table 5). In the plane, most of the stakeholders (8/10) preferences were pointed toward the positive direction of the x-axis, having less variation in their preferences of the control options, while stakeholders 5 and 10 had major deviation from the group. BCG vaccination was not among the top three ranked control options for these two stakeholders. The sensitivity analysis showed that when equal weight was given to each criterion, the ranking of the three top control options remained stable except shifting in the order between the second (OP7) and third option (OP6), indicating the robustness of the study.

## Discussion

To the best of our knowledge this is the first study to identify, evaluate and rank different BTB control options using MCDA tool based on the stakeholders' opinions and preferences in Ethiopia. Despite high prevalence of the disease in dairy cattle in the country, currently there is no BTB control and /or eradication program. The participation of stakeholders to achieve the purpose and use of participatory approaches such as MCDA are helpful to identify and evaluate BTB control options. In the present study, we generated the desired data from stakeholders who represented organizations with direct responsibilities or had specific interests in BTB prevention and control in Ethiopia.

In addition to identifying, evaluating and ranking of BTB control options, the stakeholders also identified various pre-requisite measures such as legal framework for the implementation and allied issues as an integral part of a control program prior to its application. Similarly, these measures were identified and their implications on the future prevention and control of BTB in Ethiopia were mentioned as daunting tasks by Dibaba et al. (17). In agreement to these requirements, several BTB eradication programs in many countries succeeded in reducing or eliminating the disease in cattle, by employing such multi-faceted approaches in place (10). Thus, creating enabling conditions, particularly formulation of BTB control policy and implementation guidelines, should be the primary steps in order to implement prevention and control of BTB effectively in the country. This will serve as a springboard toward initiation and implementation of the identified BTB control options in Ethiopia. The role of pertinent stakeholders and researchers would be indispensible in this regard in advising policy and decision makers by creating platforms for national level sensitization and discussion.

In the present study, nine potential BTB control options consisting of three single and six combined options were identified based on the stakeholders' preferences. Under scenario 1, the stakeholders ranked BCG vaccination as the number one control option and complementary measure to the second and third control options in cattle under Ethiopian conditions. BCG vaccination refers to vaccination of calves at 6 weeks of age. Calves are immune-competent at birth and are naturally sensitized to antigens of environmental mycobacteria at a young age. By 6 weeks of age, calves usually show a strong immunological response to such antigens. BCG vaccination of calf at birth induced a high level of immunity. However, it is recommendable to vaccinate calf at 6 weeks of age (18). Despite significant knowledge gaps regarding the real impact of BCG vaccination on the incidence of BTB and the inability to distinguish between infected and vaccinated cattle using purified protein derivative (PPD) skin test (19, 20), recent experimental studies have indicated the significance of vaccinating cattle with BCG vaccine in reducing the prevalence, progression and severity of BTB and recommended BCG vaccine as a valuable tool in the control of BTB (21, 22). The use of BCG vaccination has been encouraged because of the development of a new skin test which could differentiate *M. bovis* infected animals from BCG-vaccinated animals (i.e., DIVA role) (23). The experiment conducted in Ethiopia on the evaluation of the efficacy BCG under natural challenge model demonstrated good performance of BCG particularly in reducing the severity and dissemination of the lesion (22). However, approved commercial BCG vaccines for use in cattle are not yet present on the market.

According to the stakeholders, BCG vaccination poorly reduces the prevalence of BTB in cattle. This is likely influenced by the role of BCG in sensitizing cattle to respond to the conventional BTB diagnostic test, combined with the low level of protection of BCG vaccine in cattle compared to its relative effectiveness in humans (22). To overcome the diagnostic limitations of PPD skin test, an alternative skin test that can differentiate infected from vaccinated animals (DIVA) was developed (24). Thus, in the presence of the alternative test with DIVA role BCG vaccination would be the preferred control option for control of BTB, particularly in low and middle income countries like Ethiopia (24).

The second and the third control options involve prior application of BCG vaccination to reduce the number of positive cattle followed by test and slaughter with half and full compensation by the government, respectively. In high income countries, test and slaughter is the most preferred and a widely applied approach to control and eventually eradicate BTB, since the prevalence is low (6, 7, 10). For instance, Australia is among the few countries that eradicated BTB successfully (25) while USA is on the verge of controlling BTB through tracing of infected herds identified through meat inspection, followed by test and slaughter program (26). Many European Union countries also were successful to be recognized as officially TB free countries (27). However, BTB control is not practically feasible in low and middle income countries like Ethiopia through test and slaughter method alone due to lack of resources for rigorous testing, tracing, slaughtering of large number of positive cattle, and compensations to farmers (28).

Alternatively, as revealed by the present study, applying BCG vaccination to reduce the prevalence and progression of BTB and subsequent integration with other control options such as test and slaughter, could be a novel approach for Ethiopia which can also be adopted by other low and middle income countries where BTB is endemic and implementation of test and slaughter policy is practically challenging. To that end, the availability and accessibility of commercial BCG vaccine is critically needed. In addition, mutual understanding, acceptability and cooperation between dairy cattle owners and the government are profoundly needed particularly regarding cost recovery scheme for the implementation of the approach.

Under scenario 2 (which did not consider BCG vaccination as a feasible option), test and slaughter with full compensation by the government ranked the number one preferred control option. Indeed, this could be the most preferred and acceptable option for the cattle owner from the perespective of relatively practical applicability and lower socio-economic impacts. However, this might not be acceptable to the government, as it would be too costly vis-a-vis to other priorities of the government (29). Besides the cost implication, implementation of the test and slaughter approach involves slaughtering and culling of large number of test positive cattle, especially at the beginning of the program, raising concerns of animal welfare and loss of cattle with good milk yield. This might affect the social acceptability of the control option.

Preferably, the second (test and segregation combined with test and slaughter with government support) and third (test and slaughter with cost sharing) options, would be the preferred options in developing countries. There are compelling evidences that test and segregation method significantly reduced the incidence of BTB (30, 31). The method involves segregating test negative animals at early stage of the disease from positive reactors based on whole herd testing. This is particularly important in countries where BTB control is lacking such as in Ethiopia and when BTB control is planned for the first time. For long term surveillance and application of BTB control with this method, testing the herd of all animals >6 weeks of age annually (depending on the incidence rate) and segregating between the positive and negative reactor. For positive reactor pregnant cow and segregating calf at birth is recommended (32). In case of milk from positive reactor cows, pasteurization of milk is effective treatment to avoid public risk and economic loss associated with discarding of milk. *M. bovis* is killed at pasteurization temperature and holding time (33). Use of small scale milk pasteurization and boiling of milk would be an alternative option for smallholder dairy farmers. More specifically, the authors suggested the third control option of scenario 2- test and slaughter with cost sharing as a reliable, feasible and acceptable option from economic point of view in Ethiopia. However, this requires as well advocacy and promotion to create awareness and to convince all stakeholders, particlularly the cattle owners to actively enagage in the implementation of the control options and share the associated costs. However, the social acceptance of slaughtering animals for disease control in this era would be very unlikely since BTB is not a public health emergency and due to the presence of effective public health measures such as meat inspection and milk pasteurization. These measures are not strictly followed in developing countries like Ethiopia and control of BTB in cattle contributed to breaking the cycle of trasmission of zoonotic BTB through meat and milk consumption. Given the high prevalence of BTB in the Ethiopian dairy herds, raising awareness of the public and communities at risk such as dairy farmers about the economic and public health of the diseases by the government would support the initiation of BTB control program.

In this study, the majority (70%, *n* = 10) of the stakeholders gave the highest weight to the epidemiologic criterion (reduction in the prevalence/incidence of BTB) in evaluating the identified control options, while the social ethics criteria generally received the least weight. The epidemiologic criterion is practically important for the control and eradication of BTB. For instance, the application of test and slaughter policy is challenged mainly due to economic and animal welfare reasons, and this impact is lower when the prevalence of BTB is low (6).

The study has some limitations. The MCDA was based on the stakeholder's opinion and preferences which might result in the difference of the weighing clusters and criteria due to personal bias. In addition, some of the invited stakeholders did not participate. Future nationwide large-scale surveys involving all stakeholders from dairy farmers, academia, veterinary associations, research institutions, NGOs, commodity associations, federal and state agencies would remedy the limitation of small size of stakeholder participation. The other limitation is the absence of commercially licensed BCG vaccine for use in calves at the moment. In this study, BCG vaccine is identified by the stakeholders as a potential control option given its importance in lowering the prevalence and progression of BTB and the development of new skin test, DIVA, which could differentiate *M. bovis* infected animals from BCG-vaccinated animals.

Based on the insights obtained from this MCDA, the following important long-term stepwise approaches were suggested to initiate national level discussions and to create awareness regarding the need for BTB control and eventually work toward controlling/eradicating the disease. First, establishing a national multidisciplinary BTB organizing body/council is needed that is composed of representatives from all stakeholders that would support the government in the formulation of a national level BTB control/eradication program and implementation guidelines. Second, the economic feasibility of the top ranked control options should be assessed, and resources should be mobilized to validate the best control option in a sentinel population (i.e., applying it in selected urban and peri-urban intensive dairy farms where the prevalence of BTB is presumably high). Third, it is necessary to critically evaluate the outcomes of the control program in the selected areas and then extend the best practices to scale up the program to other regions of the country. Finally, conducting persistent surveillance and monitoring of the status of BTB across the country will be needed to develop a national database that would help in periodic evaluation of the effectiveness of the control measures put in place and taking timely corrective actions as needed. An effective and safe BCG vaccine for use in cattle is critical for this approach.

In conclusion, the study used an MCDA tool in identifying and evaluating BTB control options in Ethiopia. According to the stakeholders' preferences, calf vaccination and test and slaughter with full cost compensation by government are the best control options under a scenario that included BCG vaccination and a scenario with no BCG vaccination, respectively. Moreover, the study showed that integrating calf BCG vaccination with other potential control options, in minimizing the number of test positive cattle thereby decreasing the cost of compensation for culled/slaughtered cattle and maintain animal welfare as the most suitable BTB control option in Ethiopia that can also be used by other countries, especially low income countries.

## Data Availability Statement

The original contributions generated for the study are included in the article/supplementary materials, further inquiries can be directed to the corresponding author/s.

## Author Contributions

FG, GEA, KM, TB, MM, and GA designed the study. FG, SL, TT, ZA, BU, and GA collected and summarized the data during multi-criteria decision process. FG analyzed the data. FG wrote the manuscript. FG, GEA, KM, TB, TT, MM, RS, SL, ZA, BU, and GA critically revised and edited the manuscript and approved its submission.

## Conflict of Interest

The authors declare that the research was conducted in the absence of any commercial or financial relationships that could be construed as a potential conflict of interest.
